# Crossing vessels with suspension versus transposition in laparoscopic pyeloplasty of patients with ureteropelvic junction obstruction: a retrospective study

**DOI:** 10.1186/s12894-021-00846-z

**Published:** 2021-05-06

**Authors:** Jun Liu, Jingjun Zhang, Weinan Chen, Liulin Xiong, Xiaobo Huang, Xiongjun Ye

**Affiliations:** 1 Urology and Lithotripsy Center, Peking University People’s Hospital, Peking University, 133 Fuchengmen Inner Street, Xicheng District, Beijing, 100034 People’s Republic of China; 2 Peking University Applied Lithotripsy Institute, Peking University, Beijing, 100034 China; 3 Department of Urology, Peking University BinHai Hospital (Fifth Tianjin Central Hospital), Tianjin, 300450 China

**Keywords:** Laparoscopic pyeloplasty, Crossing vessel, Ureteropelvic junction obstruction, Hydronephrosis

## Abstract

**Purpose:**

To compare the effects of two different methods of laparoscopic pyeloplasty for the treatment of crossing vessels.

**Methods:**

From January 2016 to August 2019, 33 patients with ureteropelvic junction obstruction (UPJO) underwent laparoscopic pyeloplasty at our center, including 21 men and 12 women, ranging from 14 to 66 years of age. There were 20 and 13 cases on the left and right sides, respectively. Patients underwent laparoscopic pyeloplasty (Anderson-Hynes operation). During the operation, either a Hem-o-lok clip suspension or transposition was used to treat the crossing vessels. The double-J stent was removed 8 weeks after the operation. The clinical data of patients were collected and follow-ups were regularly performed after the operation.

**Results:**

All the crossing vessels were successfully preserved, and none of them were severed during the operation. The average operation time was 210.6 ± 58.9 min in this group and the average time to manage the crossing vessel was 8.0 ± 3.5 min, 5.9 ± 1.4 min in the suspension group, and 11.7 ± 3.0 min in the transposition group. The dilation of the affected side was 4.8 ± 1.5 cm before operation and 1.2 ± 1.3 cm 3 months after operation. The difference was statistically significant (*P* < 0.05). Follow-up to February 2020 showed no significant changes in the kidney size in all patients and hydronephrosis was relieved.

**Conclusion:**

For UPJO patients with crossing vessel compression, the method of Hem-o-lok suspension or vascular transposition can be used to relieve crossing vascular compression and improve the success of pyeloplasty.

## Introduction

Ureteropelvic junction obstruction (UPJO) is a common cause of hydronephrosis and renal dysfunction. There are several causes of UPJO, such as intraureteral stenosis, ureteral dynamic dysfunction, and ectopic vascular compression. Among them, UPJO caused by crossing vessel compression accounts for 10–28.1% of adult primary UPJO [1, 2]. Ectopic renal artery, also known as crossing vessels, refers to an extra artery of the kidney, usually entering the kidney without the renal hilum, and often is accompanied by veins. Crossing vessels usually enter the lower pole of the kidney through the ventral side of the ureteropelvic junction, where mechanical compression or traction occurs, resulting in obstruction and deformation of the local ureter, poor drainage of urine, and hydronephrosis [3]. The treatment of crossing vessels has always been controversial in clinical practice [4, 5] as these vessels can provide a part of the blood supply to the kidney; complete disconnection of the crossing vessels will cause loss of part of the renal function. Therefore, preserving the crossing vessels would mean protecting the integrity of the renal function. Several methods can be used to treat UPJO, including open pyeloplasty (OP), endoscopic pyelotomy (EP), laparoscopy (LP) and robot assisted pyeloplasty (RP). The success rate of RP is the highest, the complication of LP is lower than that of OP, the success rate of EP is relatively low, but the operation time is the shortest of all surgical methods [6]. In clinical work, the management of crossing vessels has always been a difficult challenge in laparoscopic pyeloplasty (LP).

From January 2016 to August 2019, 33 cases of UPJO with crossing vessel compression operated at Peking University People's Hospital were retrospectively analyzed to explore the treatment of crossing vessels in the LP.

## Data and methods

### General clinical data

From January 2016 to August 2019, 147 LP cases were completed in our hospital, including 33 cases (21 men; 12 women) of UPJO with ectopic renal vascular compression. All cases were selected continuously at the same time period. There were no patients with second pyeloplasty. The robot platform was not available and the OP was not performed. The average age was 32 years, ranging from to 14–66 years of age. There were 20 and 13 cases on the left and right sides, respectively. None of the patients had a history of ureter or kidney operation. The main symptoms were hypochondriac pain, increased RBC count in urine, and urinary tract infection. All patients were diagnosed with UPJO by abdominal ultrasound, CT tomography urography (CTU) and/or diuretic renal dynamic imaging before surgery. The indication of operation was based on clinical symptoms (such as recurrent hypochondriac pain and urinary tract infection), imaging examination results, and obvious obstruction of diuretic renal dynamic imaging [7]. The degree of hydronephrosis was classified based on the ultrasonographic grading of hydrophysis [8]. According to the legislation of the ethics committee, we obtained the moral declaration from the ethics committee and all patients signed the preoperative informed consent.

### Operation method

General anesthesia was administered in all patients in the 70° lateral decubitus position. The skin was incised approximately at 2 cm from the umbilicus of the affected side, and pneumo-peritoneum was established by puncture using the Veress needle. Additionally, trocar (10 mm in the left hand and 12 mm in the right hand) was placed under the costal margin of the middle clavicle and the anterior superior iliac spine. When the affected side was on the right, a 5 mm trocar was placed under the xiphoid process to lift the liver.

The process began with the opening of the lateral peritoneum on the lateral side of the colon and the colon was pushed to the inner and lower parts. When lifting the dilated anterior wall of the renal pelvis, the energetic knife was carefully used to dissect and fully expose the anterior and posterior walls of the renal pelvis and the upper ureter. The crossing vessels on the ventral side of the dilated renal pelvis must be found and carefully dissociated to prevent damage to the crossing vessels.

**Fig. 1:**
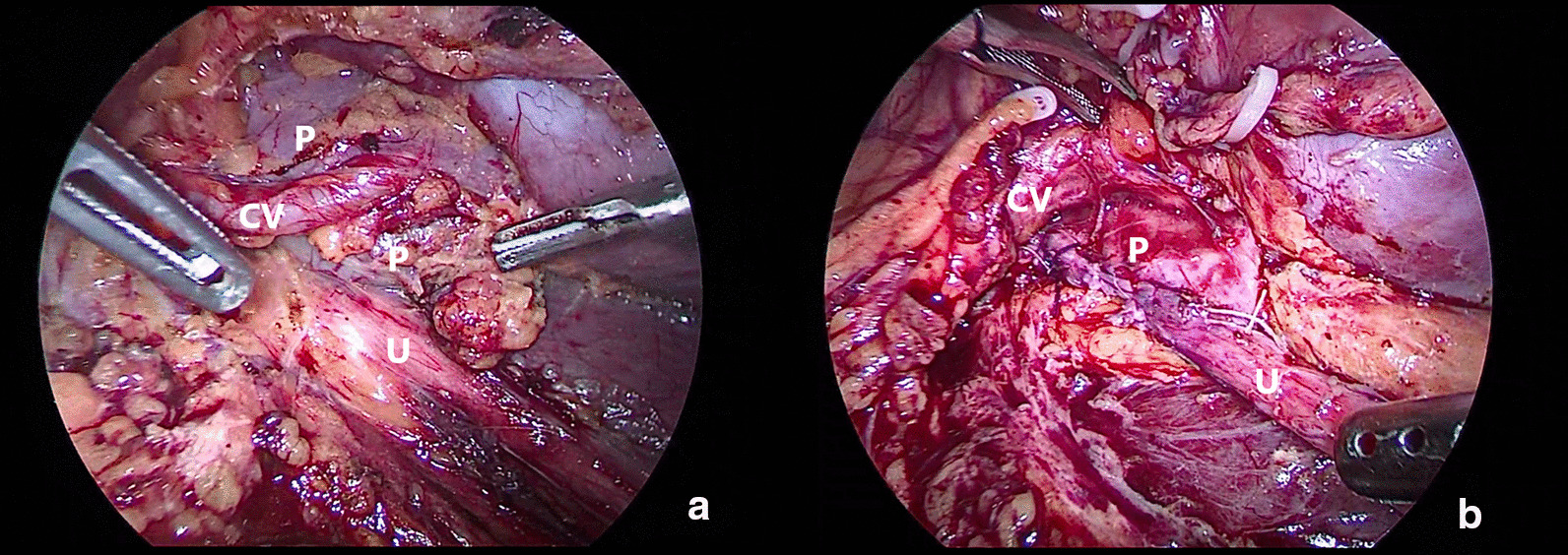
Crossing vessel suspension, **a** before LP, **b** after LP

**Fig. 2:**
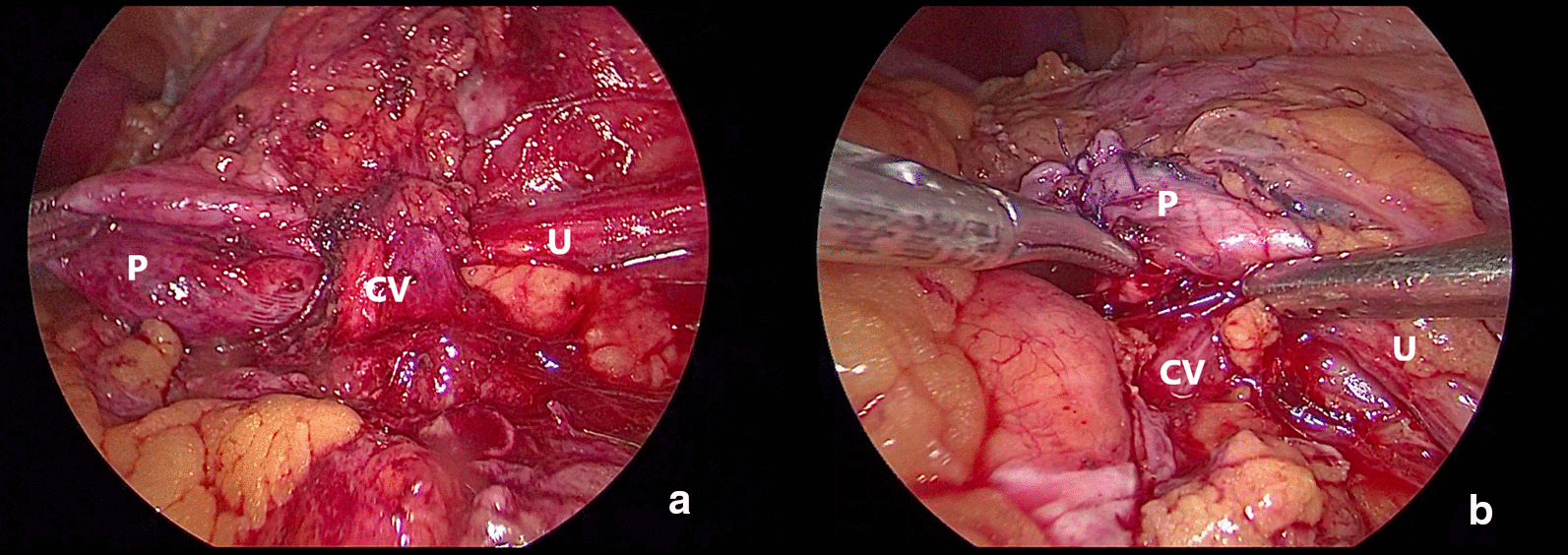
Crossing vessel transposition, **a** before LP, **b** after LP

Generally, according to the location and direction of the crossing vessels, the following two surgical methods are used. (1) Suspension with Hem-o-lok clips (Weck Surgical Instruments, Research Triangle Park, NC, USA) (21 cases in suspension group, Fig. 1): When the crossing vessel is close to the hilum (above the level of the midline of the lower pole of the kidney) and in a tortuous shape, 2–3 Hem-o-lok clips can be used to clamp the fibrous connective tissue on the surface of the crossing vessel and then fix it on the perirenal fat sac above the hilum. Subsequently, the ureteropelvic junction (UPJ) could be exposed clear manner. Attention must be paid not to damage the crossing vessel during clamping. (2) Transposition of the crossing vessel (12 cases in transposition group, Fig. 2): When the crossing vessel is at the level of the lower pole of the kidney (below the level of the middle line of the lower pole of the kidney), transposition of the crossing vessel is feasible due to the lower position and the greater tension of the crossing vessel. First, the inferior pole of the kidney is exposed with an energetic knife, the lower pole of the kidney is picked up, and the crossing vessels are carefully dissociated, in order to be completely separated from the UPJ. After the ureteropelvic junction was dismembered, the crossing vessel was transferred to the dorsal side of the anastomosis and fixed on the fascia of the psoas major muscle above the anastomosis with suture to prevent the compression from forming again.

Subsequently, the, conventional dismembered laparoscopic pyeloplasty was performed, the pelvic and ureteral posterior walls were anastomosed first with 4–0 Vicryl (Johnson & Johnson Inc, New Brunswick, NJ), a 6F double-J stent was inserted, and the ureter was sutured intermittently. In the anterior wall, the redundant pelvis was closed with continuous suture by 4–0 Vicryl, a drainage tube was placed, the lateral peritoneum was closed, and the operation was completed.

### Data collection and follow-up

The patient's medical records, including the operation time, time required to manage the vessels during the operation, estimated blood loss during the operation, drainage tube removal time after the operation, hospital stay time, and postoperative complications were collected. Due to the economic factors and China's medical insurance policy, not all patients can afford the cost of diuretic renal dynamic imaging. Therefore, the success criteria of these patients are complete clinical relief of abdominal pain and improvement of hydronephrosis by B-ultrasound. If the patient's pain is not relieved or B ultrasound still indicates that hydronephrosis is not improved, it means that the surgery failed. During surgery, all patients underwent insertion of double J stent, which was removed approximately 8 weeks after the surgery. The improvement of the hydronephrosis before and 3 months after the operation was evaluated by abdominal B-ultrasound. All patients were followed up for more than 6 months, and changes in renal size were recorded before and 6 months after the operation.

### Statistical analysis

SPSS (version 13.0) software (SPSS, Inc., Chicago, IL) was used for data analysis. Student’ s t-test was used to compare the numerical variables; Fisher’s exact test was used to compare the classified variables. *P* values < 0.05 were considered statistically significant.

## Results

All of the patients underwent successful preservation of the crossing vessels and none of them were severed during the operation. There were 5 cases of mild hydronephrosis, 15 cases of moderate hydronephrosis, and 13 cases of severe hydronephrosis (Table 1). In this group, 24 patients were confirmed to have crossing vessel compression by preoperative imaging diagnosis, and an additional 9 patients were found to have crossing vessels during the operation. Among them, 29 cases exhibited heterotopic arteries and 4 cases were accompanied by heterotopic arteries and veins. The average operation time was 210.6 ± 58.9 min in the whole group, 203.8 ± 61.8 min in the suspension group, and 222.5 ± 56.8 min in the transposition group. The mean treatment time of crossing vessels was 8.0 ± 3.5 min, that of suspension group was 5.9 ± 1.4 min, and that of the transposition group was 11.7 ± 3.0 min. The average volume of blood loss was 55.8 ± 25.6 mL. The average time of drainage tube removal was 4.6 ± 2.0 days and the average hospital stay was 11.4 ± 3.0 days (Table 2). In the transposition group, 1 patient exhibited slight urinary leakage and the drainage tube remained until the 9th day after the operation. The pathology of the stenosis of UPJ in all patients was chronic inflammation.

**Table 1:** Characteristics of patients and degree of hydronephrosis

Items	Suspension team (n = 21)	Transposition team (n = 12)	Total (n = 33)	*P* value
Age (years), ${\overline{\text{x}}} \pm {\text{s}}$	33.5 ± 13.4	31.9 ± 11.1	32.9 ± 12.2	0.513
Male/Female, n	14/7	7/5	21/12	0.222
Side (left/right), n	12/9	8/4	20/13	0.281
Degree of hydronephrosis				
Mild, n (%)	3 (14.3)	2 (16.7)	5 (15.2)	0.29
Moderate, n (%)	9 (42.9)	6 (50.0)	15 (45.5)	
Severe, n (%)	9 (42.9)	4 (33.3)	13 (39.4)

**Table 2:** Pre-operative-, intra-operative-, postoperative-, and follow-up- data of the two groups

Items	Suspension team (n = 21)	Transposition team (n = 12)	Total (n = 33)	*P* value
Operative time/min	203.8 ± 61.8	222.5 ± 56.8	210.6 ± 58.9	0.798
Crossing vascular management time/min	5.9 ± 1.4	11.7 ± 3.0	8.0 ± 3.5	0.004
Blood loss/ml	45.2 ± 36.0	74.2 ± 85.3	55.8 ± 58.4	0.182
Drainage time/d	4.3 ± 1.2	4.8 ± 2.4	4.6 ± 2.0	0.495
Hospitalization time /d	11.3 ± 2.1	11.4 ± 3.7	11.4 ± 3.0	0.967
Pre-operative hydronephrosis/cm	5.0 ± 1.6	4.6 ± 1.6	4.8 ± 1.5	0.487
Post-operative hydronephrosis/cm	1.8 ± 1.0	2.0 ± 1.8	1.2 ± 1.3*	0.269
Pre-operative length of kidney/cm	11.5 ± 1.3	11.4 ± 1.2	11.4 ± 1.8	0.720
Post-operative length of kidney/cm	11.2 ± 1.1	11.7 ± 1.3	11.5 ± 1.2#	0.291

*Compared with preoperative hydronephrosis (*P* < 0.05), #Compared with preoperative renal length, *P* > 0.05

The hydronephrosis before and 3 months after the surgery were compared using abdominal B ultrasound as the standard to evaluate the effectiveness of the operation. There was a significant difference (P < 0.05) observed in the entire group, wherein the average hydronephrosis was 4.8 ± 1.5 cm before the operation and 1.2 ± 1.3 cm 3 months after the operation. Among them, the average hydronephrosis of the suspension group was 1.8 ± 1.0 cm 3 months after the operation, and that of the transposition group was 2.0 ± 1.8 cm 3 months after the operation. There was no significant difference in the degree of pyelocele between the two groups (*P* = 0.269).

The changes in renal length before and 6 months after the operation were compared by abdominal B-ultrasound as an indirect index to evaluate whether the crossing vessels were effectively preserved. There was no significant difference between the two groups (*P* > 0.05). All patients were followed up until February 2020, with an average follow-up of 17 months, ranging from 6 to 28 months. The size of the kidneys in all patients had no significant change compared with that before operation and hydronephrosis was relieved.

## Discussion

Crossing vessel compression is one of the common causes of UPJO, mostly related to congenital development. UPJO caused by crossing vessel compression accounts for 10–28.1% of adult primary UPJO [1, 2]. The majority of the symptoms were pain or discomfort on the affected side of the waist and some patients were treated for hydronephrosis upon routine physical examination. Ectopic renal vessels (also known as renal vagal vessels) are mainly divided into ectopic renal arteries and ectopic renal veins (ectopic arteries are more common), which are usually located on the ventral side of the ureteropelvic junction and do not enter the kidney through the renal hilum. Additionally, some of them directly enter the lower pole of the kidney, with an incidence of about 6.3% [1].

There are different surgical methods for the treatment of crossing vessels. Hellström et al. [9] proposed an operation method for a vascular hitch (Hellström operation) as early as 1949, which is considered the classic operation to deal with UPJO caused by crossing vessel compression. At present, Pesce et al. [10, 11] is known to still recommend the Hellström operation by laparoscopy for UPJO with crossing vessels. It is presumed that the operation is relatively simple; on the other hand, it can decide whether to further perform pyeloplasty according to the severity of the obstruction. Also, the advantage of LP is that it can obtain better esthetic outcome (the smaller scar) than that of OP [12]. Zhang [13] reported that eight cases of UPJO with crossing vessels underwent Hellström operation by retroperitoneal laparoscopy, all of which were successful. Based on fully dissecting the crossing vessels in the upper ureter and obstruction, the crossing vessels were embedded with 4–0 absorbable sutures and fixed on the anterior wall of the renal pelvis. This operation method ensured the blood supply to the kidney and completely resolved the problem of external compression of the ureteropelvic junction. Notably, indications need to be strictly observed.

Other authors have used the method of vascular transposition to deal with UPJO with crossing vessels. Boylu et al. [14] reported that 48 cases of UPJO with crossing vessels underwent laparoscopic pyeloplasty with robot assistance, of which 18 cases were successfully transposed. According to the author's opinion, it is necessary to judge whether crossing vessels cause actual compression to the pelvic canal according to the condition during the operation. Villemagne found that furosemide challenge test is a feasible method to determine whether there is UPJO during operation [15].

Pesce [10] arrived at a conclusion regarding the method of how to judge whether a crossing vessel is the direct cause of UPJO. He observed the effective peristalsis of UPJ and the rapid passage of urine from the renal pelvis by a diuretic test in 111 patients during the operation and judged whether to perform the Hellström operation. The report stated that the success rate of this method is 98% and only one patient received suspension of operation with a poor effect. It is worth noting that crossing vascular compression is not the only factor that causes UPJO. It is equally important to resect the obstructed part of the UPJ and reconstruct the junction. In most cases, ureteral stenosis or decreased peristalsis function occurs in the compression segment. Even if the external compression is relieved, dynamic obstruction still occurs in the ureter. The data from Ellerkamp’s study showed that there was no significant difference in the pathological characteristics of ureteral smooth muscle fibrosis, muscle hypertrophy, and inflammation between the UPJO patients with and without vascular compression.

Taken together, we suggest that the treatment of crossing vessels should be determined according to the position and direction of the ectopic vessels. In short, when the crossing vessels are close to the renal hilus (above the level of the lower pole midline of the kidney), the method of blood vessel suspension can be adopted (Fig. 1). When the crossing vessels are at the level of the lower pole of the kidney (below the level of the lower pole midline of the kidney), the method of crossing vessel transposition can be adopted (Fig. 2).

The average time of the crossing vessels management was 5.9 ± 1.4 min in the suspension group and 11.7 ± 3.0 min in the transposition group (*P* < 0.05). This is because the crossing vessels in the transposition group directly enter the lower pole of the kidney and the vessels are relatively short; therefore, they need to be completely free from the root of the lower pole of the kidney to the starting part. Subsequently, they should be fixed on the fascia of the psoas major muscle above the anastomosis with suture.

The obvious limitation of our study is the small number of patients. Furthermore, we know that there are several factors that can affect the final outcome of pyeloplasty: methods, approaches, techniques, as well as the manner of suturing and stenting. In addition, retrospective studies inevitably lead to case selection bias. In order to reduce the bias of the two groups of patients, only the patients who underwent laparoscopic pyeloplasty by the same surgeon were chosen.

In conclusion, the method of Hem-o-lok clips suspension or vascular transposition can be used to relieve the crossing vascular compression for UPJO patients with crossing vessel compression at the same time of pyeloplasty, according to the location and direction of the ectopic vascular, so as to further improve the success rate of pyeloplasty. It is simple and reliable to use Hem-o-lok clips to suspend ectopic blood vessels, which has a certain value in clinical applications.

## Data Availability

The datasets used and analyzed during the current study are available from the corresponding author on reasonable request.

## Abbreviations

UPJ: Ureteropelvic junction
UPJO: Ureteropelvic junction obstruction
LP: Laparoscopic pyeloplasty
CT: Computed tomography
CTU: Computed tomography urography
